# Peritoneal Window With Omental Interposition as a Prophylactic Measure to Prevent Post-Renal Transplant Lymphocele: A Pilot Study

**DOI:** 10.7759/cureus.68406

**Published:** 2024-09-01

**Authors:** Deerush Kannan, Mathisekaran Thangarasu, Rajesh Paul, Pravin Meenashi Sundaram, Deepak Raghavan

**Affiliations:** 1 Urology, Apollo Hospitals, Chennai, IND; 2 General Surgery, Apollo Hospitals, Chennai, IND

**Keywords:** prophylaxis, morbidity, peritoneal fenestration, lymphocele, renal transplant

## Abstract

Introduction: Renal transplant is considered to be the most optimum treatment option for chronic kidney disease. One common post-operative complication that can compromise the graft function is lymphocele. Despite the technical advances, the incidence of lymphocele is not negligible. Here, we propose the outcomes of peritoneal window and omental interposition as a prophylactic measure to prevent lymphocele occurrence.

Methods: This was a single-centre prospective study conducted at a tertiary care hospital, between June 2021 and June 2023. The study included patients more than 18 years of age who underwent renal transplants. Both live-related and deceased renal transplant recipients were included. The primary endpoint focused on the incidence of symptomatic post-transplant lymphocele necessitating interventional treatment within six months of follow-up.

Results: Out of 50 patients who underwent renal transplants during the study period, only one patient developed lymphocele in the postoperative period.

Conclusion: Prophylactic peritoneal window with omental interposition serves as a promising technique to prevent post-renal transplant lymphocele formation.

## Introduction

Renal transplantation is the optimal treatment for kidney failure, associated with a lower risk of mortality [1] and improved quality of life compared with those on dialysis [2]. The incidence of surgical complications after renal transplant has decreased over the last two decades, from as high as 30% to less than 6% currently, which is attributed to the refinement of operative techniques in kidney procurement, recipient bed preparation, and techniques in transplantation [3]. Despite the technical advances, one common complication faced by the recipient surgeon is lymphocele formation, which has an ill effect on the graft kidney causing compression of the graft and graft dysfunction [4]. The incidence of lymphocele in post-renal transplant scenarios varies between 5% and 33% [5]. Various causes are proposed as a cause for lymphocele formation, and no single technique is universally recognized to overcome this complication [6]. The ultimate treatment option left with the urologist when a recipient presents with lymphocele causing graft dysfunction is by creating a peritoneal window either by an open or laparoscopic method [7,8]. Though there are randomized control trials [9] and a few studies having comparative data on peritoneal fenestration as a prophylactic measure to prevent lymphocele formation [10], data on omental interposition are less. Here, we report the first prospective study on omental interposition at the graft hilum through a peritoneal window to further reduce the incidence of lymphocele.

## Materials and methods

This was a single-center prospective study conducted at a tertiary care hospital between June 2021 and June 2023, involving the population presenting for renal transplant. This study was conducted according to the ethical guidelines of the Declaration of Helsinki and its amendments. The study was approved by our Institutional Ethics Committee. All patients participating in this study provided informed consent.

The study included patients who were more than 18 years of age. Both live-related and deceased renal transplant recipients were included. Eligible patients were informed about the study protocol and requested to provide written informed consent to participate. All patients, including those who received either the left or the right kidney, were enrolled in the study. Patients who were unable to comply with the follow-up requirements of the study, who were unwilling to participate in the study, recipients of combined transplantations (e.g., pancreas-kidney transplantation, liver-kidney transplantation), and those who underwent second or third renal transplant surgery were excluded from the study.

The other data that were shown interest included body mass index(BMI), primary diagnosis for renal failure, cardiovascular history, smoking status, presence of diabetes mellitus, hypertension, type of renal replacement therapy before transplant, duration of PD before transplantation, the presence of a peritoneal dialysis (PD) catheter during surgery, and history of PD infections in the past, history of previous abdominal surgery, native nephrectomy, pelvic radiotherapy, ABO incompatibility, and the requirement for anti-T cell therapy in case of rejection.

Steps of surgery: Recipient surgery commenced with a Gibson incision, facilitating access to the retroperitoneum by medially sweeping the peritoneum. Vascular dissection entails the individual ligation of small tributaries of the external iliac vein (EIV), external iliac artery (EIA), and internal iliac artery (IIA). Using a monopolar cautery at a low setting, the fascia over the vessels was meticulously opened. Our standard practice involves preparing the EIA, IIA, and EIV for anastomosis. Lymphatic channels were not necessarily sought and ligated. Before receiving the graft kidney, warm saline wash is given.

Graft kidney retrieval was done by open or minimally invasive methods, and benching to isolate the vessels for length was done post-retrieval. Vascular anastomosis, followed by ureteroneocystostomy by modified Lich-Gregoir technique, was then performed. Meticulous hemostasis was ensured.

**Figure 1:**
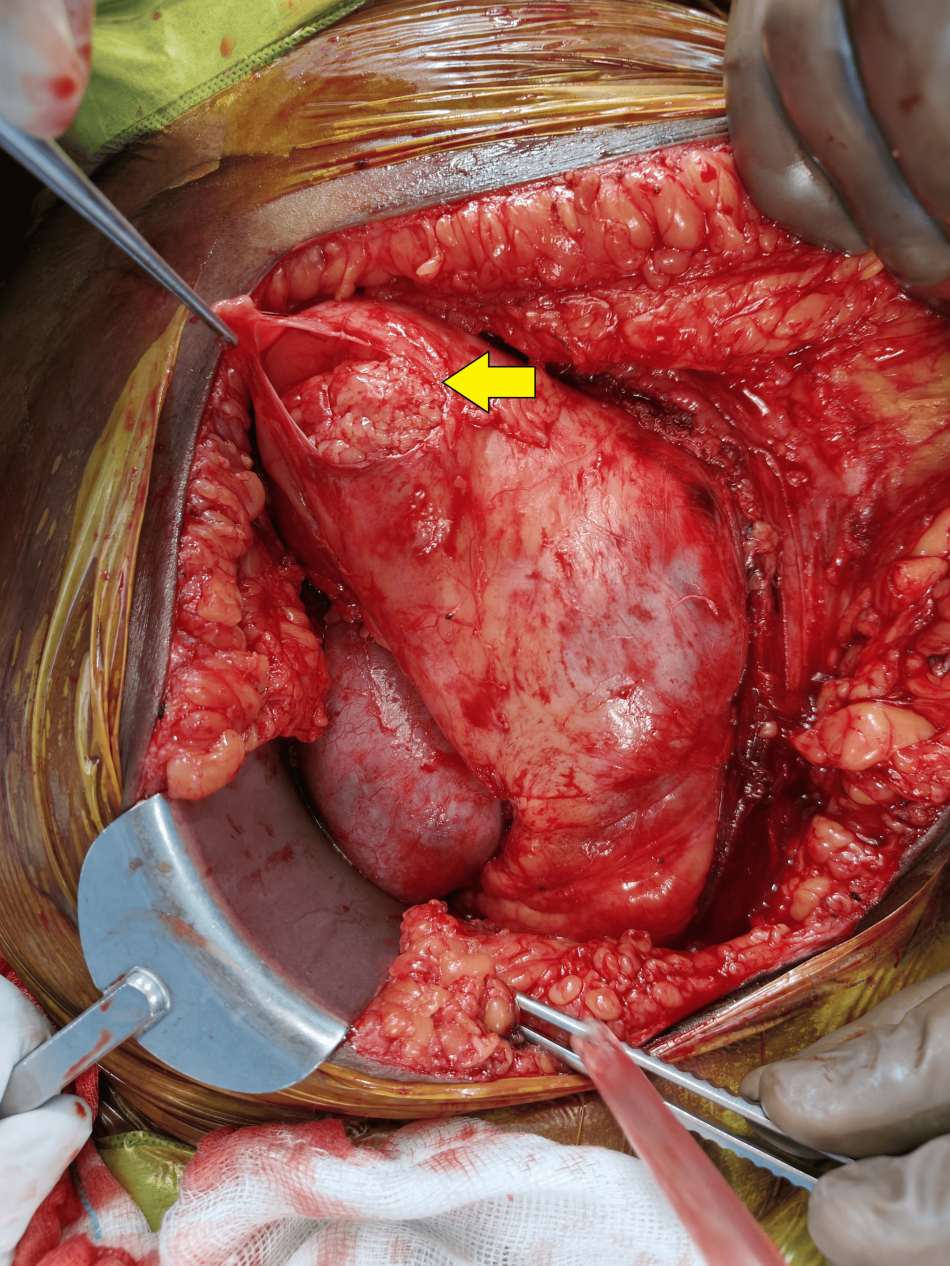
Creation of the peritoneal window

**Figure 2:**
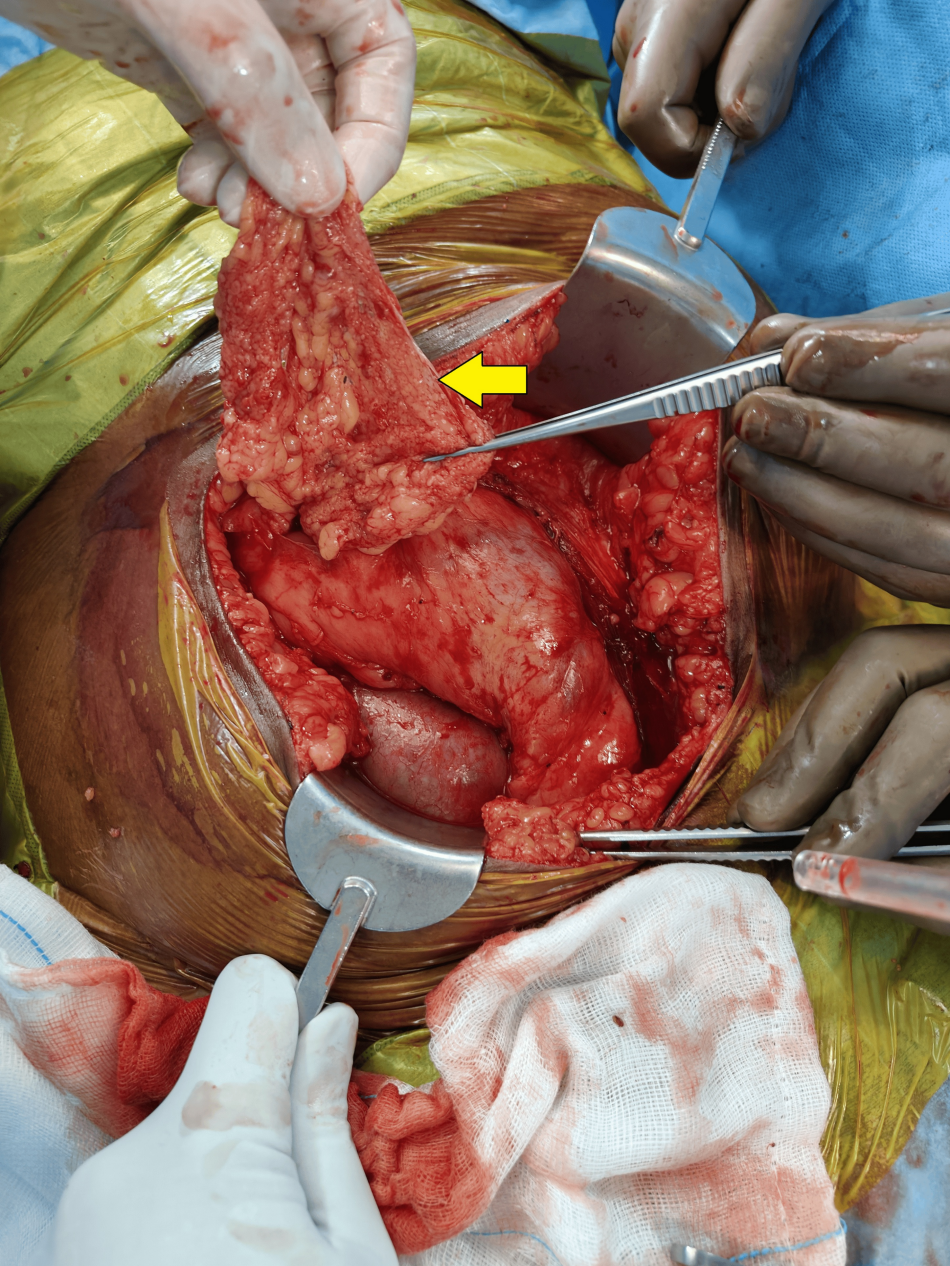
Mobilization of the omentum through the peritoneal window

**Figure 3:**
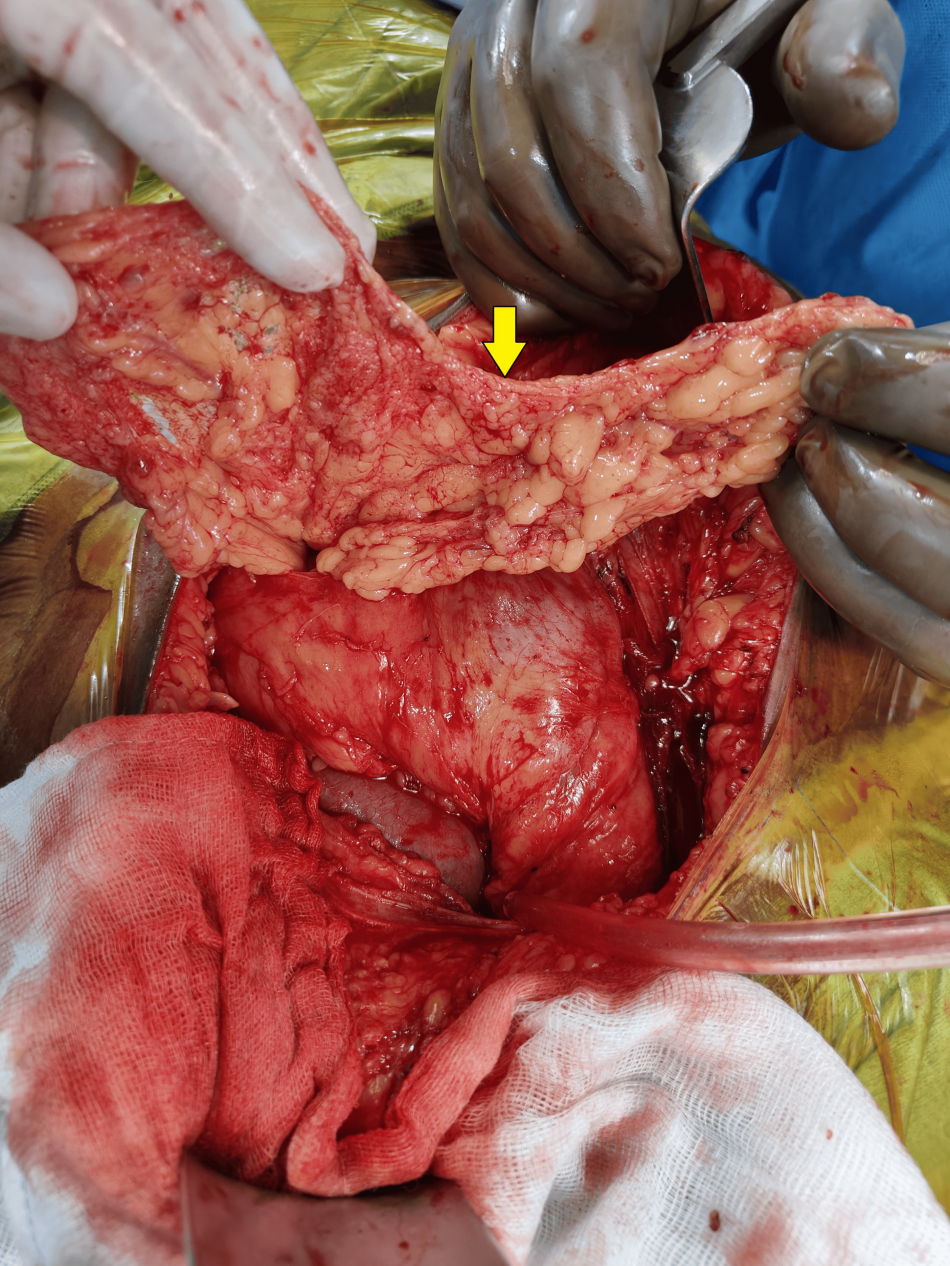
Adequately mobilised omentum

**Figure 4:**
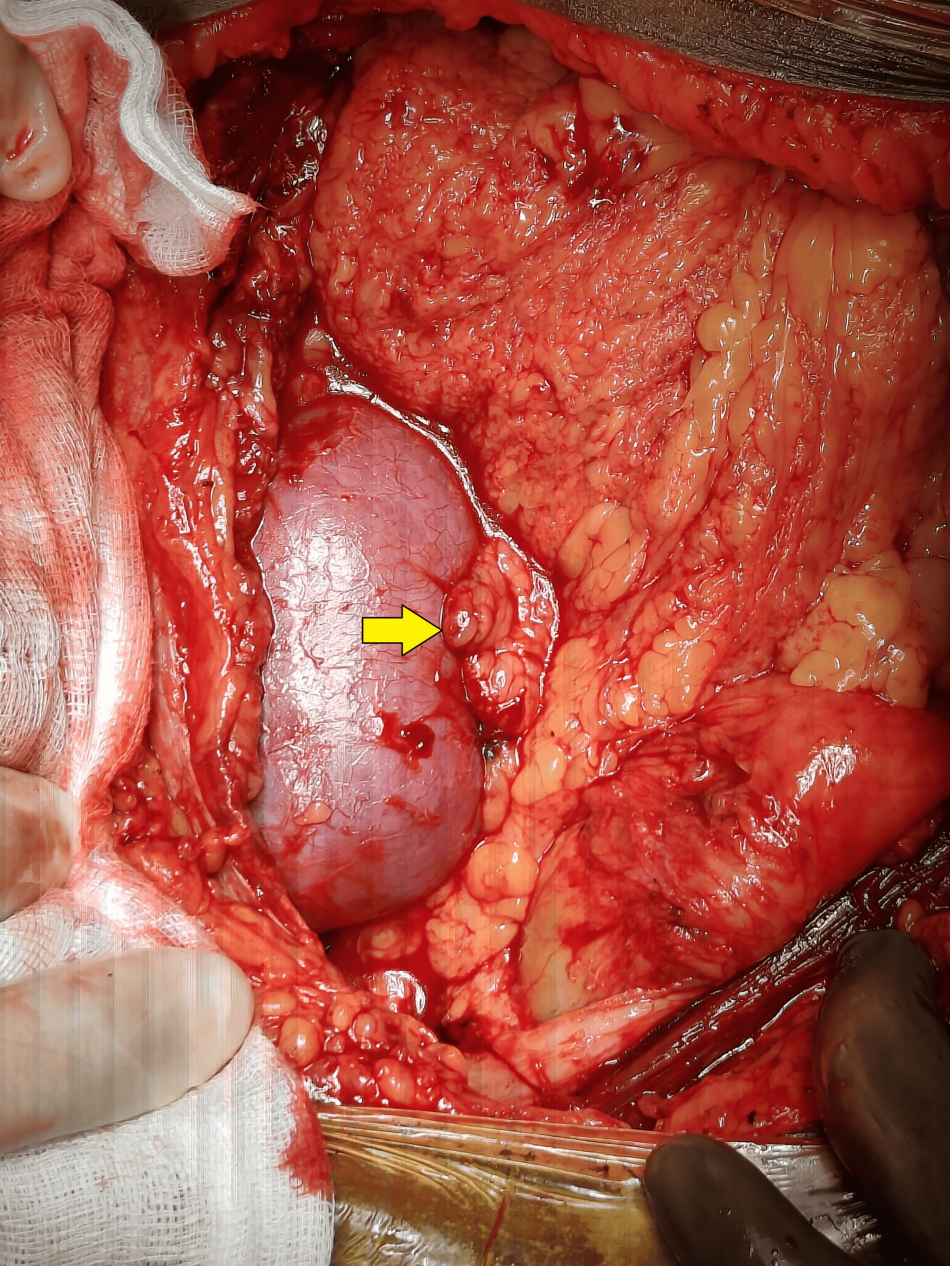
Mobilized omentum placed over the graft hilum

Following this, a 2 cm incision was made on the peritoneum, and a segment of the omentum was mobilized through this incision to facilitate and place the omentum at the graft hilum (Figures 1-4). Notably, no sutures or clips were employed to secure the omentum or maintain the patency of the peritoneal window. A 20 Fr drain was placed in all the patients. Subsequently, abdominal closure was done.

All patients underwent perioperative monitoring in a transplant intensive care unit. Subsequent postoperative follow-up visits were scheduled at months one, three, and six. Ultrasound examinations were conducted bedside during inpatient stays on the specified PODs and before drain removal. For the study's purposes, ultrasounds were repeated during follow-up visits, consistently performed by the same sonologist. Comprehensive documentation encompassed demographic details, baseline clinical data, intraoperative findings, graft function, and postoperative outcomes.

The primary endpoint focused on the incidence of symptomatic post-transplant lymphocele necessitating interventional treatment within six months of follow-up. Secondary outcomes included the duration of hospital stay, postoperative complications (such as fluid collections, burst abdomen, incisional hernia, intestinal herniation through the peritoneum, intra-abdominal abscess or collection, and postoperative ileus), and all-cause mortality. Data for these secondary outcomes were diligently recorded for all patients.

## Results

During the study period, renal transplant procedures were performed on 58 patients with a mean age of 40.87 years and a standard deviation of 11.94 years. Patients were enrolled on an accrual basis, with a majority being male (61.5%). Eight patients were lost to attrition, and eight were under antiplatelet therapy that was stopped seven to 10 days before the transplant, and none of the cadaveric recipients were on antiplatelets. Five patients underwent deceased donor transplants, out of which three were right kidneys. Among the 45 patients with live-related transplants, 38 received a left kidney, and seven received a right kidney, as mentioned in Table 1. The primary reason for choosing the right kidney from live donors was the presence of multiple vessels in the left kidney or it being an obligatory right kidney donor based on split renal function on diethylenetriamine pinta-acetic acid (DTPA). All transplants were conducted in the right iliac fossa. Three patients had previously undergone peritoneal dialysis catheter placement, with all catheters placed in the left paraumbilical region.

**Table 1:** Clinical characteristics of the cohort

Total number of patients	50
Mean Age	40.87
Male	31 (62%)
Female	19 (38%)
Living Related Donor Transplant	45 (90%)
Left Kidney	38
Right Kidney	7
Deceased Donor Transplant	5 (10%)
Left Kidney	2
Right Kidney	3

Total ischemia times varied among cases due to factors such as the build-up of the patient and delays in procuring the donor's kidney. Consequently, emphasis was placed on vascular anastomotic time. The mean vascular anastomotic time was 32 minutes, ranging from 27 to 38 minutes. Forty-five patients received anticoagulants during anastomosis before clamping the EIV. The commonly followed triple immunosuppressive regimen included mycophenolate mofetil, tacrolimus, and steroids. None of the patients were on mammalian target of rapamycin (mTOR) inhibitors postoperatively. Urinary catheters were removed on postoperative day five in 42 patients, with drain removal performed the following day. Strict criteria for drain removal included an output of less than 30 mL in the 24 hours post urinary catheter removal. Urinary catheters were removed when a 24-hour urinary output was less than 6,000 mL. Five patients had their drains removed on postoperative day seven, for whom urinary catheters were removed on postoperative day six. The remaining three patients were managed with a urostomy bag attached to the drain after discharge from the hospital because of persistent drain output. In this subset of three patients drain removal was done on POD 11, 13, and 14, respectively. It is noteworthy to note that, in this subset, two patients had a BMI of more than 30, and the other patient had a peritoneal dialysis catheter placement previously. These three patients had a drain fluid creatinine assessment done before drain removal to exclude urine leaks. Drain fluid output was also noticed to be high in two patients who had ascites.

Although ultrasounds were performed on multiple occasions, only scans conducted after drain removal were considered for statistical analysis, as patients with drains in situ did not exhibit any collections around the graft site.

All patients had an ultrasound performed at two weeks and one, three, and six months of the post-operative period. Of the entire cohort, only one patient showed signs of collection on the scan. This patient had a 9 x 2.5 cm collection along the whole length of the kidney. It was identified in one of three of the surveillance scans. However, there was no hydroureteronephrosis. It is also worth noting that three out of the 50 patients required renal biopsy for medical indications, and it was performed under ultrasound guidance without any complications despite the omental interposition.

## Discussion

Vascular dissection during bed preparation in renal transplant exposes lymphatic channels around the iliac vessels. Additionally, the retroperitoneum lacks the capacity for epithelisation. These contribute to increased lymph production with diminished reabsorption, consequently leading to lymphocele formation. Additionally, dense lymphatics around the hilum of the donor kidney are considered a significant contributing factor [11].

Potential preventative measures include individually tying small vessels around the iliac vessels during bed preparation, applying polymeric sealant, washing the graft bed with off-label povidone-iodine, prophylactic peritoneal fenestration, etc. Despite all these measures, lymphocele remains a challenging surgical complication posing a risk to graft function [12]. Prophylactic peritoneal fenestration has been studied in randomized controlled trials and has demonstrated positive outcomes [10]. While the use of omental interposition in the management of difficult lymphoceles has been described [13], their role in prophylaxis remains under-explored.

The greater omentum prevents the recurrence of lymphocele by preventing early closure of the peritoneal window. It also has an abundance of lymphatic tissue and lymphangiogenic properties. This property has made it very useful in the treatment of other lymphatic issues such as upper limb lymphedema after breast cancer treatment [14]. This study underscores the significance of the technique of the creation of a peritoneal window with omental interposition, a focus that has been relatively limited in previous investigations.

In comparison to earlier data, our study reveals a lower incidence of postoperative lymphocele of 2% [15]. Additionally, our findings suggest no significant challenges in conducting graft biopsies during the postoperative period, a point not consistently supported in the literature.

Inoue et al. described that anastomosis onto the EIA was a risk factor for symptomatic lymphoceles [16]. They also stated that the lymphatic fluid originates from the recipient’s iliac lymph trunk rather than from the graft kidney. In our series, three patients underwent graft artery anastomosis to the EIA. They did not develop lymphocele.

It has always been discussed that ischemia and reperfusion affect many regulatory systems in the renal tissue and may trigger an immune response, resulting in a distinct inflammatory reaction of the kidney graft [17,18]. In our study, the mean warm ischemia time (WIT) was three minutes and 40 seconds, and the mean vascular anastomotic time before clamp release was 32 minutes. The patient with lymphocele had WIT and anastomotic times close to the mean levels.

Two cases of burst abdomen were observed during this study. Though a direct association with peritoneal window could not be conclusively established, the multifactorial nature of this complication should be acknowledged. Both these patients had a BMI of more than 27 kg/m^2^, with poor musculature and tissue quality.

The serial follow-up ultrasound examinations revealed a trend of negligible collection in the graft bed in our study. Unfortunately, there is a lack of comparative data from other studies in this specific area, which could provide valuable insights, given the inherently lower incidence of lymphocele formation in our study when compared to others [10]. Importantly, patients with previous transplants were deliberately excluded to prevent potential data skewing. A subgroup analysis focusing on this patient population, along with BMI-based assessment, holds promise for yielding more nuanced results and identifying potential indications for the peritoneal window technique with omental interposition.

The study has limitations in that the definitive complications associated with renal biopsy post peritoneal window creation have not been studied in an extensive subset of patientsm and long-term follow-ups are needed to know the outcomes.

## Conclusions

In conclusion, the peritoneal window with omental interposition demonstrates a notable reduction in the formation of lymphoceles, consequently diminishing the necessity for re-explorations and the associated morbidity. This advantage is achieved without compromising critical surgical outcomes. The validation of these findings through extensive randomized controlled trials has the potential to establish this technique as a standard of care, particularly for a subgroup of patients identified with high risk for lymphocele formation.
